# DNA Epigenetics in Addiction Susceptibility

**DOI:** 10.3389/fgene.2022.806685

**Published:** 2022-01-25

**Authors:** Graham Kaplan, Haiyang Xu, Kristen Abreu, Jian Feng

**Affiliations:** Department of Biological Science, Program in Neuroscience, Florida State University, Tallahassee, FL, United States

**Keywords:** DNA methylation, DNA modification, epigenetics, addiction, susceptibility

## Abstract

Addiction is a chronically relapsing neuropsychiatric disease that occurs in some, but not all, individuals who use substances of abuse. Relatively little is known about the mechanisms which contribute to individual differences in susceptibility to addiction. Neural gene expression regulation underlies the pathogenesis of addiction, which is mediated by epigenetic mechanisms, such as DNA modifications. A growing body of work has demonstrated distinct DNA epigenetic signatures in brain reward regions that may be associated with addiction susceptibility. Furthermore, factors that influence addiction susceptibility are also known to have a DNA epigenetic basis. In the present review, we discuss the notion that addiction susceptibility has an underlying DNA epigenetic basis. We focus on major phenotypes of addiction susceptibility and review evidence of cell type-specific, time dependent, and sex biased effects of drug use. We highlight the role of DNA epigenetics in these diverse processes and propose its contribution to addiction susceptibility differences. Given the prevalence and lack of effective treatments for addiction, elucidating the DNA epigenetic mechanism of addiction vulnerability may represent an expeditious approach to relieving the addiction disease burden.

## Introduction

DNA cytosine methylation (mC) is a major epigenetic modification in which methyl groups are covalently bound to the 5-carbon position of cytosine bases by DNA methyltransferases (DNMTs; e.g., DNMT1, DNMT3A, and DNMT3B) (Jaenisch and Bird, 2003). Cytosine methylation occurs at both cytosine-guanine (CG) dinucleotides (i.e., mCG) and non-CG sites (i.e., mCH, where H stands for A, T, or C), with mCH being particularly abundant in neurons (Xie et al., 2012; Lister et al., 2013). Methyl-sensitive transcription factors and proteins, such as Methyl-CpG binding protein 2 (MeCP2), can bind to modified cytosine and interact with repressor and chromatin remodeling molecules to mediate gene expression (Skene et al., 2010; Clemens et al., 2020). Although cytosine methylation is considerably stable, it can also be oxidized by the ten-eleven translocation methylcytosine dioxygenases (TETs; TET1, TET2, and TET3), which convert mC into hydroxymethylcytosine (hmC). hmC can persist as a stable epigenetic modification or be further oxidized by TETs into formylcytosine (fC) and carboxylcytosine (caC). During active DNA demethylation, fC and caC are excised and replaced with unmethylated cytosine through base excision repair (BER) mechanisms (Wu and Zhang, 2014; Xu and Bochtler, 2020). Together, these processes comprise and regulate DNA epigenetic dynamics in the brain.

**FIGURE 1:**
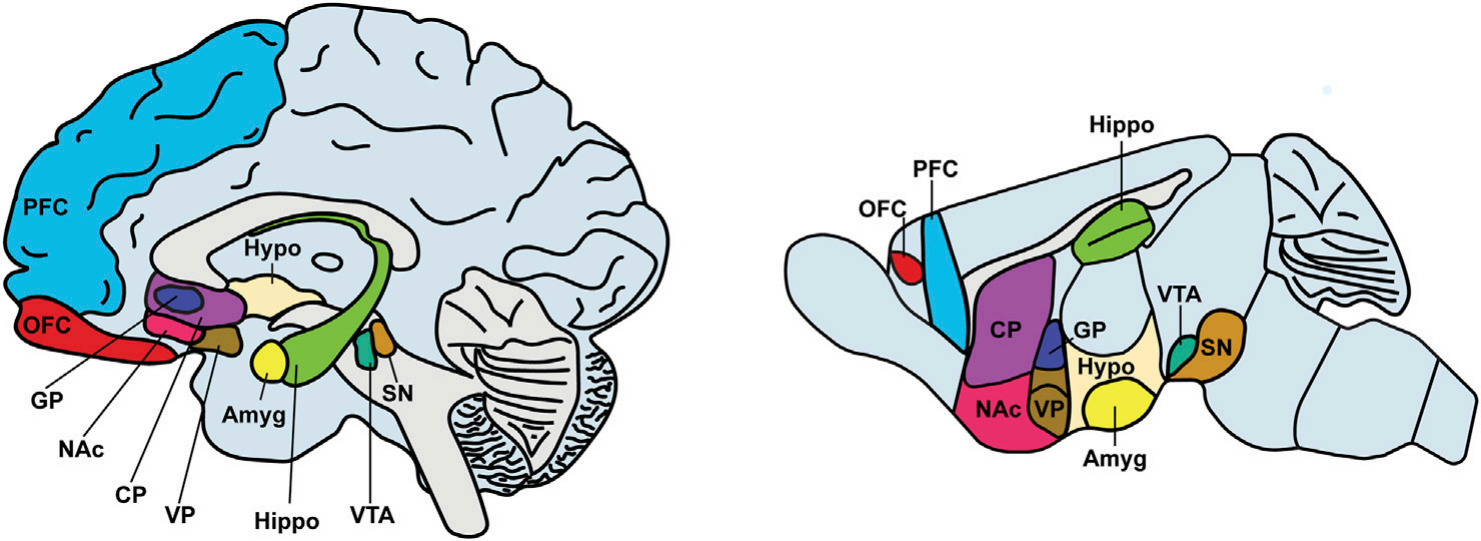
Brain regions implicated in drug addiction. The figure depicts a sketch of the sagittal sections of the human (left) and mouse (right) brains. Key brain regions engaged by drug action and implicated in addiction are highlighted with different color codes. Abbreviations: Amyg, amygdala (yellow); CP, caudate putamen (purple); GP, globus pallidus (navy); Hippo, hippocampus (light green); Hypo, hypothalamus (ivory); NAc, nucleus accumbens (pink); OFC, orbitofrontal cortex (red); PFC, prefrontal cortex (blue); SN, substantia nigra (brown); VP, ventral pallidum (dark brown); VTA, ventral tegmental area (green).

As potent forms of environmental stimuli, drugs of abuse affect the expression and activity of DNA epigenetic machinery (Figure 1; Table 1), resulting in DNA methylation and gene expression changes in the brain (Robison and Nestler, 2011; Starkman et al., 2012; Brown and Feng, 2017; Tulisiak et al., 2017; Cadet and Jayanthi, 2021). For example, dynamic changes in DNMT3A expression were observed in mouse nucleus accumbens (NAc), a key brain reward region, after cocaine administration (Anier et al., 2010; LaPlant et al., 2010). Likewise, DNMT expression is altered in multiple brain regions by alcohol (Warnault et al., 2013; Sakharkar et al., 2014; Barbier et al., 2015; Vrettou et al., 2017) or opioid use (Chen et al., 2021; Fan et al., 2021). TETs and MeCP2 are also affected by exposure to drugs of abuse, which further suggests a functional role of DNA epigenetics in addiction (Deng et al., 2010; Im et al., 2010; Feng et al., 2015; Anier et al., 2018; Jiang et al., 2021). Numerous studies have reported a global DNA methylation change in the brain after drug exposure (Table 1) and systemic administration of methionine, a methyl donor, altered the behavioral response to cocaine (Tian et al., 2012; Anier et al., 2013; Wright et al., 2015). With the advent of molecular profiling, many drug-induced DNA modification changes were found at synaptic plasticity genes, which can affect their expression and persist after cessation of drug use (Baker-Andresen et al., 2015; Feng et al., 2015; Massart et al., 2015). Therefore, DNA epigenetics contribute to the neural and behavioral adaptations associated with drug addiction and may serve as a molecular target to manipulate responding to drugs.

**TABLE 1:** List of publications on DNA epigenetic modifications in the brain in drug action.

Brain region	DNMT and/or TET evaluations	Global level and/or gene-specific DNA modification measurements	Genome-wide DNA modification profiling
Orbitofrontal Cortex			Kozlenkov et al. (2017)
Prefrontal Cortex	Ponomarev et al. (2012); Tian et al. (2012); Barbier et al. (2015); Wright et al. (2015); Hashimoto et al. (2017); Vrettou et al. (2017); Saad et al. (2019); Fan et al. (2020)	Ponomarev et al. (2012); Tian et al. (2012); Barker et al. (2013); Qiang et al. (2014); Baker-Andresen et al. (2015); Barbier et al. (2015); Wright et al. (2015); Wang F. et al. (2016); Saad et al. (2019); Fan et al. (2020); Salehzadeh et al. (2020); Iamjan et al. (2021); Vrettou et al. (2021); Yang et al. (2021)	Manzardo et al. (2012); Baker-Andresen et al. (2015); Wang F. et al. (2016)
Caudate Putamen	Vrettou et al. (2017); Saad et al. (2019)	Barker et al. (2013); Bendre et al. (2019); Saad et al. (2019); Vaillancourt et al. (2021a); Vaillancourt et al. (2021b); Vaillancourt et al. (2021a); Vaillancourt et al. (2021b); Vrettou et al. (2021)	Vaillancourt et al. (2021b); Zillich et al. (2021)
Nucleus Accumbens	Numachi et al. (2007); Anier et al. (2010); LaPlant et al. (2010); Warnault et al. (2013); Chandra et al. (2015); Feng et al. (2015); Finegersh et al. (2015); Wright et al. (2015); Anier et al. (2018); Cannella et al. (2018); Jayanthi et al. (2018); Urb et al. (2020); Vaher et al. (2020); Jiang et al. (2021)	Anier et al. (2010); Anier et al. (2013); Barbier et al. (2015); Feng et al. (2015); Massart et al. (2015); Wright et al. (2015); Cadet et al. (2017); Cervera-Juanes et al. (2017b), Cervera-Juanes et al. (2017a); Engmann et al. (2017); Anier et al. (2018); Jayanthi et al. (2018); Bendre et al. (2019); Jayanthi et al. (2020); Vaher et al. (2020); Vaher et al. (2020)	Feng et al. (2015); Massart et al. (2015); Cadet et al. (2017); Cervera-Juanes et al. (2017b), Cervera-Juanes et al. (2017a)
Globus Pallidus and Ventral Pallidum		Vetreno et al. (2020)	
Hippocampus	Liu et al. (2018); Fan et al. (2020); Zhang et al. (2020); Chen et al. (2021); Fan et al., 2021; Jiang et al. (2021)	Barker et al. (2013); Barrow et al. (2017); Fan et al. (2019a); Fan et al. (2020); Fan et al. (2021); Iamjan et al. (2021)	Sadakierska-Chudy et al. (2017)
Amygdala	Sakharkar et al. (2014); Augier et al. (2018); Sakharkar et al. (2019)	D'Addario et al. (2013); Sakharkar et al. (2019)	
Hypothalamus		Comasco et al. (2015); Barrow et al. (2017)	
Ventral Tegmental Area	Vrettou et al. (2017); Fan et al. (2019b)	Fan et al. (2019b); Maier et al. (2020); Vrettou et al. (2021)	

The table summarizes the primary publications that directly assessed DNMT and/or TET expression, global or gene-specific DNA modifications, or profiled genome-wide DNA modification landscapes after drug exposure. All citations are listed in the format of first author and year of publication. “Brain region” denotes the brain area in which results were obtained. “DNMT and/or TET evaluations” includes studies where changes in DNA methyltransferases and/or TET methylcytosine dioxygenases were examined. “Global level and/or gene-specific DNA modification measurements” includes studies where global DNA modification levels and/or gene-specific DNA modifications were assessed. “Genome-wide DNA modification profiling” includes studies in which drug-induced DNA modifications were assessed using a genome-wide or whole-genome approach (i.e., microarray or next-generation sequencing).

While addiction is a chronically relapsing neuropsychiatric disease (Nestler, 2005; Koob and Volkow, 2016), it only occurs in a fraction of individuals who use drugs of abuse recreationally, highlighting the need to understand factors that contribute to such addiction susceptibility (Grant et al., 2017; Vsevolozhskaya and Anthony, 2017). Despite the growing evidence of DNA modifications in drug action, a majority of these studies utilized two-way designs to compare drug treatment groups with drug-naïve controls, and individual variation in behavioral responding to drugs of abuse is not examined. Mounting evidence highlights numerous factors, such as life style/experience, cell type, development/ageing, and sex that not only contribute to addiction susceptibility, but also are highly associated with alterations of DNA modifications. We therefore hypothesize that DNA modifications underlie the neural and behavioral adaptations associated with inter-individual differences in addiction susceptibility. Though it remains a largely open topic of inquiry, here we aim to review and explore the plausible links between DNA epigenetics and individual variations in addiction vulnerability across several dimensions, which should expand our understanding of the molecular basis of addiction susceptibility.

## Addiction Susceptibility Differences and DNA Modifications

### Differences in Addiction Susceptibility

Inter-individual differences in behavior, which are prevalent in humans and animals, affect how individuals respond to their environment. Environmental factors, such as maternal care quality, have long been appreciated for their role in shaping individualized behaviors through persistent gene expression changes in brain areas involved in emotional regulation (Meaney, 2001). Likewise, stochastic events that occur across the lifespan can lead to the development of individualized behaviors, which is exemplified in monozygotic twins and inbred rodent strains carrying the same respective genetic background. The ability of individualized behaviors to emerge from identical genetic machinery is evolutionarily advantageous, as it permits adaptation to dynamic environmental and social conditions (Trillmich et al., 2018). For example, the behavior of inbred mice becomes progressively divergent across their lifespan when they are raised in enriched environments**.** The opportunity to explore and engage with novel environmental conditions leads to inter-individual differences, such as in patterns of exploratory behavior, social behaviors, and cognitive traits. Underlying these behavioral changes are morphological, physiological, and molecular adaptations throughout the brain. Over time, differences in experience can thus drive the development of distinct behavioral phenotypes within members of the same population (Torquet et al., 2018; Kempermann, 2019).

In addition to their evolutionary and ecological implications, neural plasticity changes may also lead to inter-individual differences in maladaptive behaviors. This is especially relevant to drug use, since drugs of abuse commonly affect brain dopamine systems and hijack the neural mechanisms regulating the experience of natural reward and reinforcement learning. Under normal circumstances, forming associations between rewarding stimuli and the contexts that signal their availability is adaptive. With repeated exposure, the cues and contexts associated with rewards can elicit behavioral activation, even in the absence of the rewarding stimuli (McDougall et al., 2014). This specific type of behavioral plasticity is mediated by dopaminergic neurons in the ventral tegmental area (VTA), which release dopamine in the NAc of the ventral striatum during encounters with rewards (Schultz et al., 1997). These transient increases in dopamine facilitate behavioral plasticity by strengthening associations between rewarding stimuli and the environmental contexts that precede their availability (Schultz, 2016). As a result, subsequent exposure to reward-predictive cues and contexts can activate brain motivational circuitry and elicit an internal anticipatory state and goal-directed behavioral responding (Cheer et al., 2007; Mannella et al., 2013; Pezzulo et al., 2014).

Indeed, drug-induced plasticity changes in brain motivational systems are believed to underlie the transition from recreational drug use to addiction (Robinson and Berridge, 1993). Specifically, repeated drug use can lead to increased sensitivity, or sensitization, of brain motivational circuitry in some, but not all, drug users, causing drugs and their associated contexts to exert potent influence over goal-directed behaviors, even after prolonged abstinence (Robinson and Berridge, 2001). Evidence for this perspective is demonstrated by an interesting phenotype which emerges in a subset of rodents during successive rounds of reward learning tasks. When rodents are trained to associate the presentation of a cue (e.g., a light) with the delivery of a reward (e.g., alcohol), most subjects learn to respond to the cue by approaching the location in which the reward will be presented (“goal-trackers”). However, a subset of subjects fail to develop this association and instead exhibit a compulsion to approach and engage with the cue itself (“sign-trackers”) (Flagel et al., 2009). The sign-tracking phenotype is thought to reflect a maladaptive behavioral program which emerges during reward learning and increases the likelihood of addiction. In agreement, “sign-trackers”, relative to “goal trackers”, display greater motivation to work for drug rewards (Saunders and Robinson, 2011), preference for drug over alternative rewards (Tunstall and Kearns, 2015), and are more susceptible to relapse after prolonged abstinence (Saunders and Robinson, 2010). Additionally, during reward learning training, reward-predictive cues maintain phasic dopamine release in the NAc of “sign-trackers”, but not “goal-trackers” (Flagel et al., 2011). Similarly, individuals with a history of cocaine dependence also exhibit increased dopamine release in the NAc and striatum in response to cues associated with cocaine (Childress et al., 1999; Goldstein et al., 2009; Young et al., 2014). Considered together, these reveal that addiction susceptibility involves hyper-sensitivity of brain motivational circuitry to drugs and drug-related cues.

Additional behavioral traits that vary between individuals have been shown to affect addiction vulnerability. For example, novelty-seeking, or the tendency to seek out intense emotional sensations and experiences, can predict drug use (Bardo et al., 1996). Rodents classified as high novelty-seekers exhibit increased behavioral sensitivity to stimulant drugs and a greater tendency to relapse after prolonged abstinence (Arenas et al., 2016). The neural mechanisms underlying novelty-seeking differences have been linked to the prefrontal cortex (PFC), which regulates decision-making, behavioral inhibition, emotionality, and the response to stress via diverse intra-cortical and subcortical connections (Koob and Volkow, 2016). High novelty-seeking rodents exhibit many signs of PFC dysfunction, including increased risk-taking behaviors, behavioral impulsivity, and exacerbated glucocorticoid secretion during acutely-stressful situations (Kabbaj, 2006; Flagel et al., 2014). A greater propensity for novelty-seeking and risk-taking behaviors increases the likelihood of initiating and escalating drug use (Cain et al., 2005), while a heightened stress response can increase negative affective states during drug withdrawal and the likelihood of relapse (Koob, 2008). Furthermore, it was found that acute amphetamine increases dopamine release in the human NAc, and subsequent identical doses of amphetamine administered 2 weeks and 1 year later produced a greater dopaminergic response. Moreover, the dopamine signal spread to dorsal striatum, and the magnitude of this effect was positively correlated with subjects’ novelty-seeking assessments (Boileau et al., 2006). Thus, novelty-seeking, though indicative of PFC dysfunction, also involves heightened and prolonged sensitivity to the dopamine-stimulating effects of drugs of abuse.

Considered together, the natural capacity for behavioral plasticity can lead to individual differences in responding to drugs of abuse. Some behavioral phenotypes, including high novelty-seeking and excessive attribution of attentional resources to drugs and drug-related contexts, confer particular susceptibility to excessive drug use and addiction.

### DNA Modifications and Addiction Susceptibility

Despite the in-depth characterization of behavioral phenotypes that are at greater risk for addiction, the molecular underpinnings of these behavioral adaptations remain elusive. DNA modifications have long been acknowledged to underlie activity-dependent gene expression changes in the brain and long-term plasticity (Chen et al., 2003; Martinowich et al., 2003; Miller and Sweatt, 2007; Feng et al., 2010). With the appreciation of neural plasticity as the mechanistic link between drug action and drug-induced behavioral adaptations (Nestler, 2001), DNA epigenetics has emerged as an intermediary in this capacity (Brown and Feng, 2017). Determining factors that lead to differential establishment of DNA epigenetic modifications in brain reward regions should therefore provide insight into the neural basis of addiction susceptibility.

To date, though still limited in number, a few studies have demonstrated the potential role of DNA epigenetics in addiction susceptibility. For example, rats that have been trained to self-administer methamphetamine (METH) displayed differences in drug taking after drug infusions were subsequently paired with electric shocks. The electric shocks were introduced after 20 days of shock-free METH self-administration to model the negative effects of chronic METH use in humans. Though all rats readily self-administered METH during the shock-free period, only some of them maintained drug seeking in the presence of electric shocks. Using hydroxymethylated DNA immunoprecipitation followed by sequencing (hMeDIP-seq), between-group differences in hmC were revealed in the NAc of these shock-resistant (more addicted) rats compared to rats that stopped taking drug after electric shock exposure. Specifically, differential hmC occurred in gene bodies of eight potassium channel genes of the shock-susceptible rats, whereas shock-resistant rats were similar to controls (Cadet et al., 2017). Since all subjects self-administered METH prior to the introduction of electric shocks and were treatment-naïve before self-administration, these addiction behavioral phenotype-specific DNA modification changes may represent epigenetic signatures underlying addiction susceptibility differences.

In non-human primates, DNA methylation is also associated with individual differences in alcohol use disorder (AUD) susceptibility. Over the course of 1 year of daily access to alcohol, divergent patterns of drinking behaviors emerged. Based on patterns of daily alcohol intake and blood alcohol levels, male rhesus macaques were classified into low/binge drinkers and high/very high drinkers. Whole-genome bisulfite sequencing was then applied to assess DNA methylation in the NAc. A discrete set of differentially-methylated regions (DMRs) were identified between low/binge drinkers and high/very high drinkers and correlated with average daily alcohol intake (Cervera-Juanes et al., 2017b). A majority of alcohol dose-dependent DMRs were found within gene bodies and regulatory regions of genes involved in synaptic plasticity. Importantly, the methylation levels at these sites were correlated with subjects’ average daily alcohol intake and may be associated with gene expression change. Furthermore, some alcohol dose-dependent DMRs were revealed that distinguished the low/binge drinker from alcohol naïve or high/very high drinking groups. These included DMRs linked to genes encoding ion channels, cell adhesion molecules, and cAMP, NF-κβ and Wnt signaling pathways (Cervera-Juanes et al., 2017a). Together, the results suggest that DNA methylation remodeling in the brain may be an index of AUD susceptibility, and DNA methylation changes in discrete genes in the NAc may influence specific alcohol drinking phenotypes associated with variable risks for developing AUD.

The phenotypic DNA methylation changes observed in these studies suggest that addiction susceptibility may involve differential expression of DNA epigenetic modifying enzymes. In support of this notion, DNMT and TET were found to be transcribed differently in the amygdala in a preclinical model of AUD susceptibility. When allowed to choose between alcohol and a natural reward (sucrose), about 15% of rats will select alcohol over sucrose. These alcohol-preferring rats also display features of human alcoholics, including increased motivation to work for alcohol and compulsive alcohol seeking in the presence of aversive stimuli (Augier et al., 2018). By using a custom Nanostring array, decreased expression of *Tet1* and *Dnmt1* was detected in the amygdala of alcohol-preferring rats, which implies downstream phenotype-specific differences in DNA modifications. However, a caveat to this interpretation and other studies mentioned here is that subjects classified as susceptible to addiction also consistently consume more drug over time than their less-susceptible counterparts. As drug exposure itself has been known to induce time-dependent DNA epigenetic changes in brain reward regions, the drug intake differences over time may therefore have contributed to differential methylation in the susceptible animals (Massart et al., 2015; Brown and Feng, 2017; Ploense et al., 2018; Sakharkar et al., 2019). However, a recent study (Vaher et al., 2020) provided evidence of drug dose-independent DNA epigenetic changes in addiction vulnerability. This was demonstrated in rats that were pre-screened for differences in exploratory behavior in a familiar environment [i.e., high and low explorers (Mällo et al., 2007)]; a behavioral phenotype that is similar to novelty-seeking and is concomitant with differential responding to substances of abuse (Alttoa et al., 2007). Cocaine intraperitoneal injections administered passively by an experimenter (i.e., each animal received identical drug doses) differentially regulated the expression of *Dnmt3b* and *Tet3* in the NAc of high- and low-exploring rats, leading to phenotype-specific DNA methylation signatures at genes implicated in addiction (Vaher et al., 2020). Furthermore, these differences were only present after cocaine exposure, demonstrating that DNA epigenetic changes associated with addiction behavioral phenotypes may be drug dose independent.

Taken together, among the growing number of studies on DNA epigenetics in drug action, a few have assessed its role in addiction susceptibility differences. Though it remains to be determined how much susceptibility variation may be attributed to DNA methylation changes, the evidence is beginning to establish that addiction susceptibility differences are associated with discrete DNA methylation events in brain reward regions. This was also supported by the finding that the strength of memory for natural rewards is regulated by DNA methylation in VTA dopamine neurons (Day et al., 2013). The study of within-group and individual differences in animal models of addiction may thus represent an expeditious approach to uncovering mechanisms of addiction etiology.

## Brain Cell Type-Specific DNA Modifications and Addiction Susceptibility

### Brain Cell Type-Specific Roles in Addiction

Numerous brain regions are involved in addiction, including cortical (e.g., orbitofrontal cortex (OFC), prefrontal cortex (PFC), and cingulate cortex), striatal (e.g., dorsal and ventral striatum, including NAc), and other basal ganglia (e.g., substantia nigra, globus pallidus, and subthalamic nucleus) structures within the mesolimbic system (e.g., ventral tegmental area) (Figure 1). Many of these brain regions have been found to undergo DNA epigenetic changes after drug exposure (Table 1). Furthermore, among the brain circuits involved in drug addiction (Dreyer, 2010; Koob and Volkow, 2010; Robison and Nestler, 2011; Bobadilla et al., 2017; Volkow and Boyle, 2018; Luscher et al., 2020), some have been shown to be associated with addiction susceptibility. For example, a disrupted connectivity between PFC and striatal regions has been identified in persons with substance use disorders (Tomasi and Volkow, 2013). In rodents, it was found that activation of the PFC projections into dorsal regions of the striatum promotes compulsive optogenetic self-stimulation of VTA dopamine neurons (Pascoli et al., 2015; Vickstrom et al., 2021). In addition, by using fMRI imaging, addiction vulnerability was further found to be associated with reduced functional connectivity among ventromedial caudate, OFC and ventromedial PFC (Ersche et al., 2020).

One challenge to studying the molecular underpinnings of drug addiction *in vivo* is the heterogeneous cell populations in the brain, with each cell type presumably carrying out distinct functions in addiction. To exemplify this concept, we will take medium spiny neurons (MSNs) in NAc to demonstrate how two morphologically indistinguishable neuron subtypes that are intermingled together play contrasting roles in addiction. Centrally located in the brain reward circuitry and highly implicated in motivated behavior and addiction, the NAc receives inputs from both dopaminergic neurons of the VTA and glutamatergic neurons of the hippocampus, amygdala, and PFC. Approximately 95% of NAc neurons are MSNs, which can be classified into dopamine D1 receptor-expressing and dopamine D2 receptor-expressing MSNs (D1-MSNs and D2-MSNs, respectively). The remaining neuron types, such as cholinergic and GABAergic interneurons, comprise 1-2% of NAc neuronal cell types (Lobo and Nestler, 2011). Though NAc D1-and D2-MSNs have similar numbers and morphologies, they belong to different circuit pathways; namely, the direct pathway and the indirect pathway, respectively. The excitatory direct pathway originates from D1-MSNs in NAc and projects to the globus pallidus (GP) and substantia nigra pars reticulata (SNr), whereas the inhibitory indirect pathway originates from D2-MSNs, projects to and terminates at the external segment of the GP (Gerfen and Surmeier, 2011; Lobo and Nestler, 2011). Extensive literature has reported the distinct roles of D1-and D2-MSNs in drug-induced responses (Kravitz et al., 2012; MacAskill et al., 2014; Khibnik et al., 2016). For example, it was shown that NAc D1-MSNs displayed decreased membrane excitability and increased frequency of miniature excitatory postsynaptic currents following repeated cocaine exposure, while D2-MSNs exhibited an attenuated miniature excitatory postsynaptic currents frequency with no change in excitability (Kim et al., 2011). Stimulation of D1-MSNs induced persistent cocaine reinforcement, while inhibition of D1-MSNs projecting to the ventral pallidum blocked drug seeking (Bock et al., 2013; Pardo-Garcia et al., 2019). Furthermore, inhibition of NAc D2-MSNs enhanced motivation to obtain cocaine, as reflected by lever-pressing behavior during drug self-administration in rodents (Bock et al., 2013). Chemogenetic or pharmacological manipulation of D1-or D2-MSN activity also differentially affected behavioral responding to repeated alcohol intake, further supporting the distinct roles of these two neuron subtypes in drug addiction (Kravitz et al., 2012; Cheng et al., 2017).

Consistent with the contrasting functions of D1-and D2-MSNs, these two neuron subtypes also have unique molecular signatures that mediate their respective roles in addiction behavior (Lobo et al., 2006; Heiman et al., 2008). Moreover, the same gene may play different roles in D1-and D2-MSNs. For instance, deletion of *TrkB*, the receptor of brain-derived neurotrophic factor (BDNF), in D2-MSNs suppresses cocaine reward, while the same manipulation in D1-MSNs generates the opposite behavioral consequence (Lobo et al., 2010). In another example, the early growth response 3 (*egr3*) molecule, which binds to the promotor area of several cocaine-regulated genes, was found to execute opposite roles in NAc D1-and D2-MSNs during cocaine-induced behavioral responding (e.g., conditioned place preference, locomotor activity) (Chandra et al., 2015). The N-methyl-d-aspartate (NMDA) receptor, a major receptor subtype for the neurotransmitter glutamate, was also shown to function differently in NAc D1-and D2- MSNs after chronic alcohol or cocaine exposure (Joffe et al., 2017). Importantly, *Dnmt3a*, the DNA methyltransferase that was previously shown to mediate cocaine action (LaPlant et al., 2010), was decreased selectively in D1-MSNs, but not D2-MSNs, following a 24 h withdrawal from seven daily intraperitoneal cocaine administrations (Chandra et al., 2015). While DNA methylome profiling has not yet been reported in D1-or D2-MSNs, DNA methylation changes were revealed at a three-dimensional DNA loop site that was strengthened in NAc D2-, but not D1-MSNs after cocaine administration (Engmann et al., 2017). Considered together, these highly indicate the plausible contribution of cell type-specific DNA modifications in addiction etiology.

### Brain Cell Type-Specific DNA Modifications

Pronounced brain cell type-specific differences in DNA modifications have been observed, which also appear to be brain region dependent (Luo et al., 2018; Liu et al., 2021). Recent evidence has demonstrated methylation differences between neurons from distinct regions of the human brain, such as dorsolateral PFC, anterior cingulate cortex, hippocampus, and NAc (Rizzardi et al., 2019). Brain region-specific differences in neuronal mCG were reported to be associated with open chromatin and enriched with brain region-specific and activity-dependent transcription factor binding motifs. This further suggests that cell type-specific methylation works in concert with chromatin states to regulate gene expression in a brain region-specific manner. Moreover, mCG signatures in NAc were highly distinct from other brain regions, which may reflect the relative homogeneity of NAc neuronal cell types (i.e., GABAergic MSNs), whereas cortex and hippocampus consist of more heterogeneous neuron subtypes. Differential DNA methylation between neuron subtypes also demarcates gene promoters and other regulatory elements with cell type-specific functions (Kozlenkov et al., 2014; Kozlenkov et al., 2016). Cortical glutamatergic and GABAergic neurons, the major excitatory and inhibitory neuronal cell types, respectively, are depleted of mCG in and around the transcription starting sites (TSS) of genes with cell type-specific functions. mCG levels in these regions inversely correlate with gene expression. Moreover, cell type-specific mCG depletion in TSS-distal regions overlaps with enhancers with known functions in the brain, while predicted enhancers for peripheral tissues are hypermethylated in each neuron subtype. In addition, mCH plays important roles in neuronal cell type-specific gene expression regulation. Neuronal mCH was found to be most abundant in gene bodies and intergenic regions, with substantially lower levels detected in gene promoters (Guo et al., 2014; Chen et al., 2015; Kozlenkov et al., 2016). Like mCG, specific mCH patterning in these genomic contexts distinguishes cortical GABAergic from glutamatergic neuron subtypes (Clemens and Gabel, 2020). Gene body mCH accumulates at low-abundance differentially expressed genes in cortical GABAergic, glutamatergic, and NAc GABAergic neurons, suggesting that mCH in this context fine-tunes neuron subtype-specific patterns of gene expression. Notably, compared to mCG status or chromatin accessibility, differences in neuronal mCH across various brain regions appear to be better correlated with corresponding gene transcription differences (Mo et al., 2015; Kozlenkov et al., 2016; Stroud et al., 2017; Kozlenkov et al., 2018; Rizzardi et al., 2019).

Growing evidence also indicates a role for hmC in neuronal cell-specific functions. Neuronal hmC accumulates at gene bodies of actively-transcribed genes, poised and active enhancers, and exon/intron boundaries at both CG and CH contexts (Wen et al., 2014; Mellen et al., 2017; Marshall et al., 2020). Pronounced differences in global hmC have been detected between cerebellar granule vs. Purkinje cells, cerebellar Purkinje vs. hippocampal granule cells (Jiang et al., 2015), and cortical GABAergic vs. glutamatergic neurons (Kozlenkov et al., 2018). Although gene body hmC is consistently correlated with gene expression in neurons, hmC at flanking regions of active enhancers appears to be neuronal cell type-specific. For example, in GABAergic neurons from human cortex, hmCG is enriched at enhancers for GABAergic neuron-specific genes and depleted at enhancers for glutamatergic neuron-specific genes. The observed differences in hmC may reflect a later activation of GABAergic cortical neuron-specific enhancers, consistent with the protracted developmental trajectory of cortical GABAergic interneurons during early postnatal brain development (Kozlenkov et al., 2018). Together, these findings demonstrate a broad range of functions of hmC in neurons and are well situated in an emerging pattern of hmC at enhancers and actively-expressed genes in numerous human tissue types (Cui et al., 2020).

In addition to the DNA modification differences between neuronal cell types, distinct methylomes of glial cell types have been detected. Comparing whole-genome DNA methylation profiles between neurons (positive staining of NeuN, a mature neuron marker) and non-neuronal cells (NeuN negative, presumably most were glial cells) suggested that cell type-specific CG-DMRs were localized to functional regions of the genome, while mCH, which is nearly depleted in non-neuronal cell types, was considered the primary neuron-specific DNA modification (Lister et al., 2013). Analyses of human glial methylomes have found that CG methylation changes occur at non-coding and intergenic regions, which overlap with enhancers of genes with glial cell-specific functions (Kozlenkov et al., 2014). Interestingly, glial cell type-specific DNA modifications appear to be conserved during evolution. For example, the *QK1* locus, which encodes an RNA binding protein involved in myelination, is hypermethylated in cortical neurons and hypomethylated in oligodendrocytes, the glial cell type that establishes myelin in the central nervous system. This pattern of differential methylation at the *QK1* gene between neurons and oligodendrocytes was observed in both humans and non-human primates, suggesting that oligodendrocyte DNA methylation was conserved during evolution (Jeong et al., 2021). Likewise, methylation differences between cortical neurons and astrocytes, another major glial cell type, appear to be highly consistent between human and mouse (Kessler et al., 2015), which further supports the conservation of the glial methylome across mammalian brain development. As sorted glial nuclei generally lack the brain region-specific DNA methylation landscape detected in neurons, these findings suggest a more uniform role for DNA epigenetic regulation in glia (Rizzardi et al., 2019).

The recent advent of single cell methylation profiling has facilitated the recognition of brain cell types at an unprecedented level (Armand et al., 2021). In a pioneering single cell brain methylome study, numerous neuronal subtypes that carry distinct methylome signatures were identified in the frontal cortex of humans and mice (Luo et al., 2017). The methylation landscape (primarily mCH) facilitated the classification of cortical neurons not only by neuron subtype, but also by cortical layer, with more specific clusters identified within a single layer. Recently, single nucleus methylome data was obtained from 45 dissected regions of mouse brain and used to create a comprehensive brain DNA methylation atlas at single-cell resolution (Liu et al., 2021). NeuN labeling was used to separate neuronal from non-neuronal nuclei, while cell subtypes were characterized based on both mCG and mCH profiling. In total, 161 cellular subtypes were recognized, which highlights the complex heterogeneity of the cell type-specific DNA methylome in the brain. Notably, GABAergic D1-MSNs could be further divided into four subtypes based on their native brain structure (NAc vs. dorsal striatum) and spatial distribution along the anterior-posterior axis, revealing that neuronal DNA epigenetic modifications may be more spatially regulated than was known. With single-cell studies of DNA methylation in the brain growing in number, assessing whole-genome hmC at single cell levels has proven challenging, particularly owing to the low abundance of hmC as compared to mC (Mooijman et al., 2016). However, given the distinct roles of hmC, it is necessary to parse its respective genomic distribution, which is indecipherable under conventional sodium bisulfite profiling methodologies (Nestor et al., 2010; Yu et al., 2012; Booth et al., 2013). A recently-developed, whole-genome, single-cell, base resolution hmC sequencing protocol has provided a promising opportunity (Schutsky et al., 2018). With the vast heterogeneity of brain cell types illustrated by single cell DNA modification profiling, we are on path to further elucidate the complex role of DNA modifications in the brain.

Though substantial progress has been made towards understanding the role of discrete brain cell types engaged in addiction, how these neural cells come to function differently among individuals and lead to variable responding to drugs of abuse remains largely unknown. Likewise, methodological advancements in single-cell DNA modification profiling have revealed the mosaicism of DNA epigenetic modifications in the brain, but how these differences give rise to broader patterns of brain activity and behavior remains elusive. Neuronal ensembles, or subgroups of cells which respond synchronously to sensory stimuli and cognitive states, may exemplify an intersection between cell-specific DNA methylation dynamics and addiction susceptibility. Exogenous influences, such as drugs of abuse, have been shown to promote the formation of neuronal ensembles in rodent brain (e.g., NAc, amygdala, and cortex), and their synchronous activation facilitates memory retrieval and stimuli-specific behavioral plasticity (Cruz et al., 2013; de Guglielmo et al., 2016; Nawarawong and Olsen, 2020; Sun et al., 2020). A recent study found that neuronal ensemble formation can be affected by DNA methylation (Gulmez Karaca et al., 2020). Over-expression of *Dnmt3a* in mouse hippocampal ensembles enhanced the retrieval of memories in fear-conditioned mice. Moreover, its over-expression in recently-activated cultured hippocampal neurons induced hypermethylation at synaptic plasticity genes, suggesting that DNA methylation mediates the strength and stability of ensemble formation. The contribution of DNA methylation to neural plasticity in neuronal ensembles will likely give rise to broader patterns of neural and behavioral adaptations, such as those that characterize addiction. This may represent a functional connection between cell type-specific DNA modifications and addiction susceptibility.

## Time-Dependent DNA Modifications and Addiction Susceptibility

### Addiction Susceptibility and Brain Development

Considering the dynamic developmental trajectory of the brain across ontogeny, disruption of one or more underlying developmental processes may predispose individuals to addiction susceptibility. In particular, maternal alcohol use during pregnancy can impart lasting changes in the brains of offspring and increase their propensity for addiction later in life (Popova et al., 2017). Epidemiological studies have revealed that the prevalence of drug and alcohol abuse in adults born with fetal alcohol syndrome disorder is substantially higher (46%) than the lifetime prevalence of alcohol (18.2%) and drug (10.3%) abuse in the general adult population (Streissguth et al., 2004; Compton et al., 2007). It is believed that the organizational deficits in brain circuitries induced by in-utero alcohol exposure increase the likelihood of addiction later in life (Bariselli and Lovinger, 2021). Rodents pre-exposed to alcohol during prenatal development exhibit changes in dendritic morphology in D1 and D2-MSNs in NAc and enhanced excitability of dopamine neurons in the VTA in adulthood (Rice et al., 2012; Hausknecht et al., 2015). These neural changes are associated with differential sensitivity to drug action, including heightened sensitivity to the anxiolytic effects of alcohol and the rewarding and behavioral-activating effects of stimulants (Barbier et al., 2008; Wang et al., 2019). Therefore, drug exposure during prenatal development can bias the cellular and molecular architecture of the brain and predispose individuals to uncontrolled drug use in adulthood.

Whereas prenatal development establishes global brain structure, postnatal brain development is associated with the refinement and maturation of neural circuitries, including increases in myelination, connectivity, and synaptic pruning. The maturation of the PFC and limbic brain structures is especially protracted and dynamically regulated by life experience. This allows the gradual acquisition of adult cognitive and emotional behaviors in accordance with an individual’s environmental needs (Spear, 2000). Drug exposure during early postnatal development can interrupt this process and increase the chances of drug dependence later in life (Crews et al., 2007). For example, the timing of alcohol use onset is thought to be an important determinant of future development of AUD. In humans, the age of initiating alcohol use in youth predicts later-life alcohol dependence, with initiation between the ages of 11 and 12, 13, and 14, or 19 and older associated with 15.9, 9, and 1% rates of alcohol dependence in adulthood, respectively, (DeWit et al., 2000; York et al., 2004). Likewise, in rats, alcohol exposure during the early adolescent period leads to increased alcohol drinking, social anxiety, and greater sensitivity to low-dose alcohol exposure in adulthood. Adolescents are generally more sensitive to lower doses of alcohol and tend to drink to intoxication more often than adults, and early-adolescent alcohol exposure may maintain adolescent-typical alcohol drinking behaviors into adulthood (Spear, 2015). Similarly, adolescence appears to be a period of marked vulnerability to nicotine addiction, with 90% of adult smokers having first used nicotine before the age of 18 (National Center for Chronic Disease Prevention, 2012). Preclinical studies have further revealed that nicotine exposure beginning in early adolescence, relative to later adolescence, leads to increased expression of nicotinic acetylcholine receptor in NAc, increased sensitivity to nicotine, and higher rates of nicotine self-administration in adulthood (Adriani et al., 2003). These effects may also generalize to other drugs of abuse, because mice exposed to nicotine during early adolescence are more sensitive to the rewarding and behavioral activating effects of cocaine, amphetamine, and morphine, but not highly palatable food (Alajaji et al., 2016). This suggests that nicotine exposure during early adolescence biases brain development towards a propensity for drug addiction in adulthood, which is consistent with longstanding notions of nicotine being a “gateway” to illicit drug use (Kandel, 1975; Levine et al., 2011).

Considered together, the dynamic course of brain development represents a protracted window of vulnerability to the deleterious effects of addictive drugs. Drug exposure in-utero and during early life can induce a lasting sensitivity to drug action that may confer increased vulnerability to addiction later in life. Therefore, a deeper understanding of the molecular consequences of drug exposure during brain development should aid in our interpretation of how prior drug experience impacts later-life vulnerability to addiction.

### DNA Modifications in Neural Development and Ageing

DNA modifications play pivotal roles in mammalian development (Smith and Meissner, 2013). In mice, neurogenesis begins at mid-gestation and declines around birth, giving way to astrocytogenesis and oligodendrocytogensis (Sauvageot and Stiles, 2002; Miller and Gauthier, 2007). DNMT1 levels increase in neural progenitor cells (NPCs) and remain elevated throughout neurogenesis (Fan et al., 2005; Ziller et al., 2018). Loss of DNMT1 results in demethylation that leads to premature astrocytogenesis (Fan et al., 2005). At E10, DNMT3B expression increases in mouse NPCs, reaches peak levels by E13, then declines to undetectable levels by E15. In contrast, DNMT3A expression increases modestly in the brain from E10 through E17, and its longer isoform was found to persist at substantial levels in adult neurons (Feng et al., 2005). Consistent with this notion, the mCH pattern, which is established by DNMT3A, demonstrates spatiotemporal dynamics in the developing brain, with mCH downregulated at neural progenitor markers and upregulated at neuronal markers in sequential order of hindbrain, midbrain and forebrain (He et al., 2020). Relative to DNMTs, TET expression increases in abundance shortly after NPC differentiation into adult neurons (Wang Z. et al., 2016). This is accompanied by hmC accumulation at gene bodies of neuronal genes, further suggesting that TETs play an important role in NPC differentiation and gene expression regulation in newly-formed neurons (Hahn et al., 2013; Wheldon et al., 2014). Together, DNA modification enzymes play an important role in embryonic brain development through spatiotemporal lineage-specific gene expression regulation.

The early postnatal period (immediately after birth) is characterized by a shift of brain methylome dynamics, with mCG and mCH levels accumulating in neurons and repressing the expression of genes involved in embryonic brain development (Colantuoni et al., 2011; He et al., 2020). Regional and age-dependent changes in hmC were observed when assessing hmC dynamics in mouse hippocampal and cerebellar neurons during early-life, adolescence/early adulthood, and middle-age (Kriaucionis and Heintz, 2009; Szulwach et al., 2011). In seven-day old mice, global levels of hmC were substantially greater in hippocampal and cerebellar neurons than in NPCs, demonstrating the rapid increase in hmC in newly-formed neurons. hmC accumulation occurred at gene bodies and enhancers of genes with tissue-specific functions and actively involved in postnatal brain development. Genes with stable hmC enrichment across ontogeny were clustered in synaptic transmission and neurodevelopment categories, while late-onset hmC enrichment occurred at genes with more general cellular functions (Szulwach et al., 2011). Recent genome-wide hmC maps in human hypothalamus also detected hmC enrichment in genes involved in neurodevelopment and synaptic plasticity (Cui et al., 2020). Together, these data imply a conserved role for hmC in postnatal brain development in mammals and highlight the importance of DNA modifications in gene expression regulation across ontogeny.

Ageing is characterized by a global decrease in DNA methylation in the brain. Age-related brain demethylation coincides with decreased synaptic density, particularly in cortex and hippocampus, which is thought to contribute to cognitive decline in older adults (Lister et al., 2013). Recent evidence suggests that environmental enrichment can attenuate age-related DNA methylation changes and promote synaptic plasticity and adult neurogenesis in the hippocampus (Zocher et al., 2021). Differential methylation induced by environmental enrichment was found to occur largely in MeCP2 binding sites, and the corresponding genes affected by these changes were highly represented in human studies of age-related cognitive decline and neurodegeneration. These findings suggest that active engagement with the environment can have lasting changes on brain functioning that are mediated by DNA methylation and protect against the deleterious effects of aging (Xiao et al., 2016). Since DNA methylation changes occur predictably with age, they have been successfully used to predict epigenetic age, which differs between individuals with the same chronological age, presumably due to environmental, lifestyle, or health factors. Compared to chronological age, epigenetic age represented by DNA methylation states at age-related “clock” CG sites has been shown to be a more accurate measurement of health status (Horvath, 2013). Discrepancies between epigenetic and chronological ages have been used to predict the overall health and longevity of an individual (Marioni et al., 2015; Chen et al., 2016). To date, few studies have assessed the effects of drug abuse on clock CG methylation. Using the Illumina Infinium 450K platform to analyze DNA modifications in neurons isolated from postmortem OFC of persons with heroin use disorder, a younger DNA epigenetic age was found in heroin users relative to matched drug-naïve controls (Kozlenkov et al., 2017). Heroin use was also associated with differential methylation at the TET3 locus, suggesting that drug-induced regulation of DNA epigenetic machinery may modulate the DNA epigenetic age of the brain. It will be intriguing to further explore the effects of substances of abuse on epigenetic age and its potential association with addiction susceptibility in the future.

Taken together, DNA epigenetic dynamics mediate gene expression changes in pre- and post-natal brain development, with time-dependent changes in DNA modifications appearing to be integral to the development and continued functioning of neurons throughout the lifespan. Life experience may leave its imprint on the brain in the form of DNA modification signatures which denote the biological state of the brain. The temporal specificity of the DNA modification landscape may underscore the vulnerability of the brain to drugs of abuse during development and ageing.

## Sex Differences in Addiction Susceptibility and DNA Modifications

### Sex Differences in Addiction Susceptibility

Ample evidence supports sex-specific differences in addiction-like behaviors (Bobzean et al., 2014; Becker and Koob, 2016; Carroll and Lynch, 2016; Becker and Chartoff, 2019). Epidemiological data demonstrate sex differences in drug type preference and different rates of substance abuse by males and females. For example, teenage and young adult males are more likely to abuse or be dependent upon marijuana or alcohol, while same-age females are more likely to abuse or be dependent upon cocaine and psychotherapeutic drugs (Cotto et al., 2010). Furthermore, females are more vulnerable to the reinforcing effects of stimulant drugs and to stress-related substance use disorders, despite males being more likely to use drugs, having higher rates of addiction disorders (Greenfield et al., 2010; McHugh et al., 2018), and oscillating more often between abstinence and drug use (Gallop et al., 2007).

In addition to human studies, investigations of sex differences in addiction have been extensively executed in lab animals. The use of animal models, such as operant drug self-administration models in rodents, not only supports epidemiological findings of sex differences in drug use from humans, but also provides the advantage of parsing specific aspects of addiction, including sensitivity to drug reward, compulsive drug seeking, and relapse-like behaviors, among others (Schuster and Thompson, 1969). Using these approaches, studies have revealed sex-specific addiction-like behaviors that may account for sex differences in addiction susceptibility. It has been demonstrated that female rats self-administer more cocaine than males when given longer access to drug, suggesting a female-specific proclivity for “binge” drug use. Acquisition of drug self-administration refers to the transition from initial to sustained drug use and is an important indicator of the transition towards addiction (Campbell and Carroll, 2000). The ratio of male rats that acquire cocaine or nicotine self-administration is higher compared with female rats, and male rats require fewer sessions to acquire self-administration. However, females self-administer more cocaine during the first few sessions once the acquisition criteria are met (Swalve et al., 2016). Furthermore, females that acquire cocaine self-administration exhibit greater preference for cocaine than males, because females choose to continue taking cocaine, even when offered alternative rewards (e.g., food) (Kerstetter et al., 2012; Perry et al., 2013; Perry et al., 2015). Sex differences during cocaine withdrawal have also been observed, with female rodents having a delayed-onset of drug-seeking relative to males. Moreover, female rats are more susceptible to stress-induced relapse after prolonged abstinence (Kerstetter et al., 2008; Anker and Carroll, 2010; Feltenstein et al., 2011; Becker and Koob, 2016). Thus, while males may be more likely to initiate drug use than females, leading to higher rates of addiction, the magnitude and persistence of drug use appear to be more severe in females with substance use disorders. Taken together, male and female rodents exhibit various differences in addiction-like behaviors that are consistent with reports in humans with substance use disorders. These differences should be considered when investigating the biological basis of addiction susceptibility.

### Sex Differences in DNA Modifications in the Brain

Though still few in number, studies have illustrated sex-specific DNA epigenetic landscapes in the brain. Understanding such DNA modification variations should provide insights into the sexually-dimorphic addiction phenotypes.

Sex differences in DNA modifications are known to exist in peripheral tissues. In-utero exposure to cigarette chemicals was found to be associated with sex-dependent DNA methylation alterations at two imprinting genes, *IGF2* and *GR*, in human fetal liver (Drake et al., 2015). It was believed that this was due to sex-specific smoking-induced alterations of the methyl donor vitamin B12. Furthermore, it was suggested that environmental factors (e.g., carcinogens, drugs) can trigger methylation changes in peripheral samples in a sex-specific manner (Lewis et al., 2019). Differential methylation was detected in the blood of male human alcoholics relative to non-alcoholic controls, with a reported vast hypomethylation across the genome (Zhang et al., 2013). Additionally, a positive correlation between recent alcohol use history and DNA methylation was revealed in lymphocyte-derived lymphoblast cells in females. The *BLCAP* (bladder cancer-associated protein) gene was identified as the most significant target of alcohol-dependent DNA methylation changes in female lymphoblasts (Philibert et al., 2012). Notably, DNA methylation states in non-neural tissue may reflect psychiatric disease vulnerabilities. For example, DNA methylation differences were observed in the cord blood of newborn males and females. The differentially methylated sites were not only widely distributed across the genome, but also highly enriched in genes involved in neurodevelopment and psychiatric diseases (Maschietto et al., 2017; Ho et al., 2018; Xia et al., 2021).

Remarkable sex differences in the fetal brain methylome have been reported, and many of these differentially-methylated loci persist in the adult cortex (Xu et al., 2014), implying that sex differences in the adult brain methylome are established early in development (Spiers et al., 2015). By compiling data from 1,408 postmortem brain samples from three published collections, numerous sex-specific differential DNA methylation sites/regions were identified (Xia et al., 2021). Among them, many sex-specific differentially-methylated genes were enriched in synaptic plasticity and neural signaling pathways, suggesting a role for DNA methylation in sex-dependent susceptibility to psychiatric disorders. Furthermore, differential methylation was revealed in postmortem prefrontal cortex of male and female alcoholic subjects (Wang F. et al., 2016). Compared to non-alcoholic controls, male alcoholic PFC was enriched with hypermethylated CG sites that predominantly occurred in gene bodies and promoters and correlated with subjects’ alcohol use history. Differential methylation in males also affected the expression of some addiction-related genes and was enriched for genetic variant sites associated with substance abuse and neuropsychiatric disease phenotypes that were identified by GWAS studies. While significant DNA methylation changes were detected in males, none were associated with AUDs in females after multiple testing correction. Though this study was largely limited by the sample size (16 male and 7 female pairs of alcoholic and control subjects), the sex-specific DNA methylation changes in alcohol abusers suggest that DNA modifications may confer addiction susceptibility differently in males and females.

Sex differences are not only detected in the methylome, but also observed in the expression of DNA modification enzymes in the brain, which may further contribute to sexually-dimorphic risk of addiction. For example, relative to males, female rats have significantly higher levels of DNMT3A in the amygdala in the first 2 weeks after birth, which indicates a time window when DNA methylation may have different impacts on the transcriptome between males and females (Kolodkin and Auger, 2011; Chareyron et al., 2012). Additionally, female rodents also express higher levels of MeCP2 within the developing amygdala, and a transient decrease of MeCP2 disrupted social behaviors only in males, but not females (Kurian et al., 2007).

In addition to sex-dependent DNA epigenetic differences, DNA methylation was shown to maintain sex differences in the brain. While the developing brain is destined for a female phenotype, it is masculinized by gonadal hormones during the perinatal critical period. It was found that gonadal hormones function through suppression of DNMT enzymes, which can release masculinizing genes from repression. Therefore, inhibition of DNMTs or conditional knockout of *Dnmt3a* led to male sexual behaviors in female rats (Nugent et al., 2015). A study also investigated the contribution of testosterone to sex differences in DNA methylation and revealed hundreds of genes that were differentially methylated in the striatum of adult but not neonatal rats. It was further found that the effects of testosterone exposure on DNA methylation were modest in neonates, but dramatically increased during adulthood, suggesting that the impact of testosterone on the brain methylome is a progressive process that becomes prominent in adulthood (Ghahramani et al., 2014).

Gonadal steroid hormones function through their respective steroid receptors, where receptor activation translocates the ligand–receptor complex to the nucleus to mediate gene expression. Malfunctioning of this signaling pathway interrupts responding for drugs. For example, deletion of the estrogen receptor 1 gene *esr1* in mice results in altered behavioral responses to cocaine, including enhanced behavioral sensitization and an increased trend of cocaine seeking (Lasek et al., 2011). In addition, intra-NAc infusions of the DNMT inhibitor RG108 caused demethylation of the *esr1* gene promoter and activation of *esr1* transcription, whereas intra-NAc infusions of an ESR1 agonist during forced cocaine withdrawal dramatically attenuated cocaine-seeking behavior in a reinstatement test. This suggests that the methylation status of *esr1* plays a functional role during the incubation of cocaine craving after prolonged abstinence (Massart et al., 2015). Furthermore, *esr1* expression levels between males and females were also found to be different during early development, which is associated with sex dependent DNA methylation at its promoter region (Kurian et al., 2010; Schwarz et al., 2010; Hodes et al., 2017). Therefore, sex-specific DNA modifications in gonadal hormone signaling pathways represent another layer of regulation in DNA modification dependent and sexually dimorphic addiction behaviors.

In sum, sex differences in DNA modifications exist not only in peripheral tissues, but also in the brain, and they appear to be established early in development and progress through the lifespan. DNA modifications maintain gonadal hormone signaling and some sex differences in the brain that may alter behavioral output. Considering the sex-dependent DNA methylome changes in response to drugs of abuse, DNA modifications are positioned as a molecular switch for sexual dimorphism in drug use behaviors and addiction susceptibility.

## Conclusion

**FIGURE 2:**
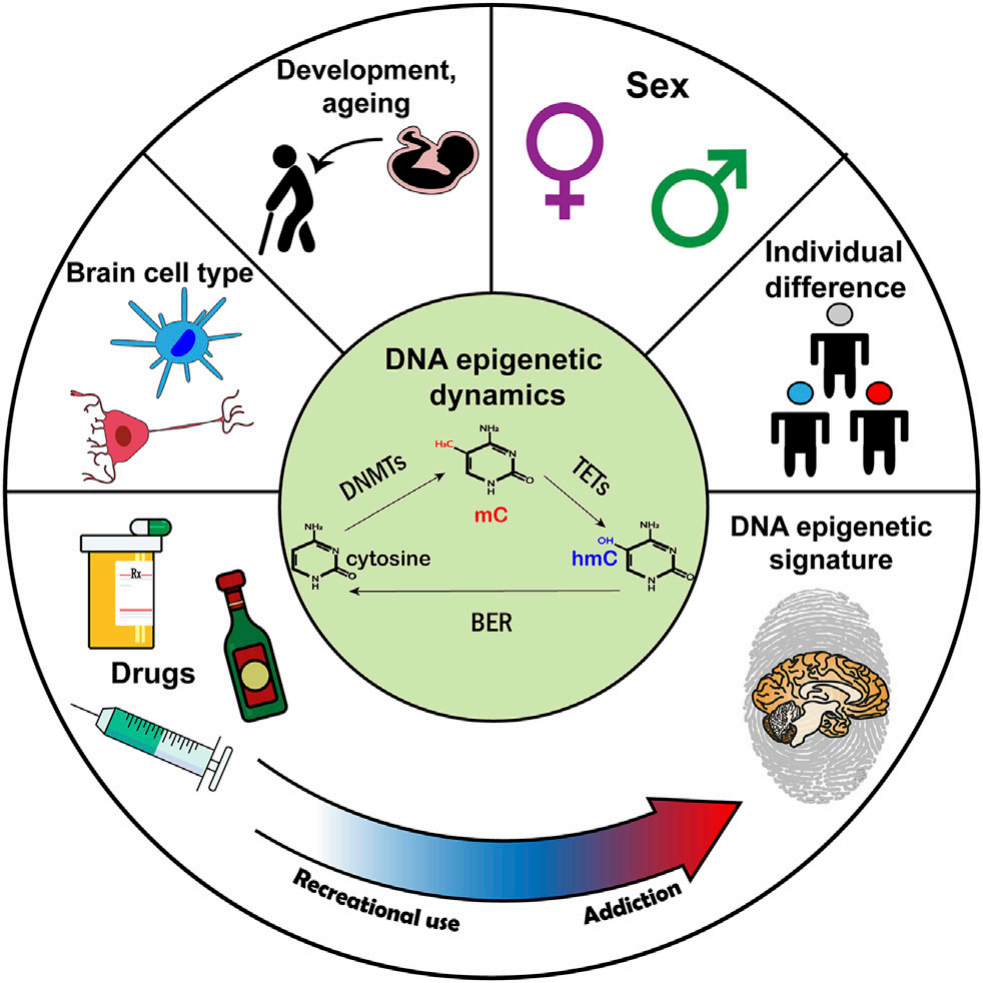
The hypothetical role of DNA epigenetics in addiction susceptibility. (Top) Factors related to addiction susceptibility that also have a DNA epigenetic basis. (Center) A schematic of DNA epigenetic dynamics. (Bottom) Addiction susceptibility is associated with DNA modification changes. Abbreviations: DNMT, DNA methyltransferase; TET, ten-eleven translocation methylcytosine dioxygenase; BER, base excision repair.

In the present review, we explored the notion that DNA epigenetic dynamics in the brain mediate inter-individual difference in addiction susceptibility. Findings from humans and animals have established that drug use affects the expression and activity of DNA epigenetic machinery, particularly in brain regions comprising the reward circuitry. However, only some individuals who use drugs recreationally go on to develop the maladaptive behavioral plasticity indicative of addiction. Growing evidence suggests that the specific propensity for addiction may have a DNA epigenetic basis, because phenotypes of addiction susceptibility are associated with unique DNA epigenetic signatures in the brain. Moreover, factors known to influence addiction susceptibility, such as sex differences and life experience, also have a DNA epigenetic basis in the brain (Figure 2). Given the remarkable heterogeneity of DNA modifications in brain cell types, determining cell-specific methylation dynamics can provide further insight into addiction susceptibility. We expect that applying cutting edge behavioral, bioinformatic, and genomic tools may greatly advance our understanding of the role of DNA epigenetics in individual differences in vulnerability to addiction.
